# CXC Chemokines Function as a Rheostat for Hepatocyte Proliferation and Liver Regeneration

**DOI:** 10.1371/journal.pone.0120092

**Published:** 2015-03-10

**Authors:** Gregory C. Wilson, Satoshi Kuboki, Christopher M. Freeman, Hiroyuki Nojima, Rebecca M. Schuster, Michael J. Edwards, Alex B. Lentsch

**Affiliations:** Department of Surgery, University of Cincinnati College of Medicine, Cincinnati, Ohio, United States of America; University of Missouri, UNITED STATES

## Abstract

**Background:**

Our previous in vitro studies have demonstrated dose-dependent effects of CXCR2 ligands on hepatocyte cell death and proliferation. In the current study, we sought to determine if CXCR2 ligand concentration is responsible for the divergent effects of these mediators on liver regeneration after ischemia/reperfusion injury and partial hepatectomy.

**Methods:**

Murine models of partial ischemia/reperfusion injury and hepatectomy were used to study the effect of CXCR2 ligands on liver regeneration.

**Results:**

We found that hepatic expression of the CXCR2 ligands, macrophage inflammatory protein-2 (MIP-2) and keratinocyte-derived chemokine (KC), was significantly increased after both I/R injury and partial hepatectomy. However, expression of these ligands after I/R injury was 30-100-fold greater than after hepatectomy. Interestingly, the same pattern of expression was found in ischemic versus non-ischemic liver lobes following I/R injury with expression significantly greater in the ischemic liver lobes. In both systems, lower ligand expression was associated with increased hepatocyte proliferation and liver regeneration in a CXCR2-dependent fashion. To confirm that these effects were related to ligand concentration, we administered exogenous MIP-2 and KC to mice undergoing partial hepatectomy. Mice received a “high” dose that replicated serum levels found after I/R injury and a “low” dose that was similar to that found after hepatectomy. Mice receiving the “high” dose had reduced levels of hepatocyte proliferation and regeneration whereas the “low” dose promoted hepatocyte proliferation and regeneration.

**Conclusions:**

Together, these data demonstrate that concentrations of CXC chemokines regulate the hepatic proliferative response and subsequent liver regeneration.

## Introduction

Loss of functional liver mass results from a variety of causes including acute liver failure, hepatic trauma, surgical resection, and transplantation. These insults trigger a regenerative response involving integrated cascades of factors controlling cell growth, angiogenesis, tissue remodeling, etc. These highly regulated signaling events lead sequentially to hepatocyte proliferation, restoration of functional liver mass, and a return to physiologic homeostasis. An important regulatory system in this process involves CXC chemokines and their receptors [1].

CXC chemokines are classified by the presence or absence of a glutamine-leucine-arginine (ELR) amino acid motif in the amino terminus, which confers receptor-binding specificity [2–4]. CXC chemokines containing the ELR motif bind to the receptors CXCR1 and CXCR2 and have been shown regulate liver repair and regeneration [5–11]. Of particular interest is the fact that this ligand/receptor system has been shown to have divergent effects on liver regeneration that is dependent upon the insult. After partial hepatectomy, in which there is loss of liver mass but little tissue injury, CXC chemokines promote liver regeneration [6,11]. In contrast, after ischemia/reperfusion (I/R) injury, in which functional liver mass is decreased but a large amount of damaged tissue remains, CXC chemokines are detrimental to the regenerative process [5,8,10].

Our previous work with hepatocytes in vitro provided evidence suggesting that the divergent effects observed in vivo could be explained by available ligand concentrations [8]. In vitro, we found that low concentrations of CXC chemokines promoted hepatocyte proliferation, whereas high concentrations of CXC chemokines resulted in increased cytotoxicity. In both cases, the effects were mediated by the receptor, CXCR2 [8]. However, whether this phenomenon occurs in vivo has not been directly tested. Therefore, in the current study, we sought to determine if ligand concentration dictates the regenerative response after hepatic I/R injury and partial hepatectomy.

## Materials and Methods

### Models of Hepatic I/R and 70% Hepatectomy

Male BALB/c and CXCR2−/− mice on a BALB/c background (Jackson Laboratory, Bar Harbor, ME) weighing 22–28 g were used in these experiments. This project was approved by the University of Cincinnati Animal Care and Use Committee and was in compliance with the National Institutes of Health guidelines.

For hepatic I/R injury, mice underwent either sham surgery or I/R. Partial hepatic ischemia was induced as described previously [12]. Briefly, mice were anaesthetized with sodium pentobarbital (60 mg/kg, i.p.). A midline laparotomy was performed and an atraumatic clip was used to interrupt blood supply to the left lateral and median lobes of the liver. The caudal lobes retained intact portal and arterial inflow and venous outflow, preventing intestinal venous congestion. After 90 minutes of partial hepatic ischemia, the clip was removed to initiate hepatic reperfusion. Sham control mice underwent the same protocol without vascular occlusion. Mice were sacrificed after the indicated periods of reperfusion, and blood and samples of ischemic lobes and non-ischemic lobes of the liver were weighed and taken for analysis.

Partial hepatectomy was performed as previously described [9]. Briefly, mice were anesthetized with sodium pentobarbital (60 mg/kg, i.p.), and a midline laparotomy was performed and 4–0 Vicryl suture (Ethicon Endo-Surgery, Cincinnati, OH) ligatures were secured around the base of the median and left lateral hepatic lobes, and the lobes were resected. Some wild-type mice were injected intravenously, via the penile vein, with recombinant murine MIP-2 and KC (Peprotech, Rocky Hill, NJ), 24 and 48 hours after hepatectomy. An identical volume of sterile phosphate-buffered saline (PBS) was used as a vehicle control. Mice were sacrificed at the indicated periods after hepatectomy, and blood and samples of remaining lobes were taken for analysis. Liver/body weight ratio was determined, and normalized to the pre-hepatectomy liver/body weight ratio.

For all experiments, euthanasia was performed by pentobarbital overdose followed by thoracotomy consistent with the recommendations of the Panel on Euthanasia of the American Veterinary Medical Association.

### Exogenous MIP-2/ KC Administration

Treatment doses were established for intravenous administration of exogenous chemokines to normal, unmanipulated mice and mice undergoing partial hepatectomy and sham operation. One treatment group received “high” dose MIP-2 (0.015 ng/g) and KC (0.24 ng/g) that replicated the serum levels of these chemokines observed 48 hours after I/R injury [8]. The second treatment group recieved “low” dose MIP-2 (0.0002 ng/g) and KC (0.006 ng/g) determined by multiplying the high dose by the ratio of observed tissue levels of MIP-2 and KC 48 hours after hepatectomy vs I/R. An identical volume of sterile phosphate-buffered saline (PBS) was used as a vehicle control. For experiments in which mice were treated with exogenous chemokines, treatments were administered at time 0 and every 24 hours thereafter with the last dose occurring 24 hours prior to sacrifice—mice sacrificed at 48 hours received injections at time 0 and 24 hours and those sacrificed at 72 hours received injections at time 0, 24, and 48 hours.

### Blood and Tissue Analysis

Blood was obtained by cardiac puncture for serum analysis. Liver content of MIP-2 and KC was assessed by enzyme-linked immunosorbent assay (ELISA). Liver samples were weighed and immediately placed in 10 volumes (wt/vol) of a protease inhibitor cocktail containing 10 nmol/L ethylenediaminetetraacetic acid, 2 mmol/L phenylmethylsulfonyl fluoride, 0.1 mg/mL soybean trypsin inhibitor, 1.0 mg/mL bovine serum albumin, and 0.002% sodium azide in isotonic PBS, pH 7.0. Tissues were disrupted with a tissue homogenizer, and lysates were incubated at 4°C for 2 hours. Samples were clarified by 2 rounds of centrifugation at 12,500*g* for 10 minutes at 4°C. ELISA reagents for these CXC chemokines were obtained from R&D Systems (Minneapolis, MN). Liver tissues were fixed in 10% neutral-buffered formalin, processed and then embedded in paraffin for light microscopy. Sections were stained with hematoxylin and eosin for histological examination.

### Proliferating Cell Nuclear Antigen (PCNA) Staining

Immunohistochemical staining for PCNA was performed on paraffin-embedded liver tissue with anti-PCNA antibody using DakoCytomation ARK kit (Dako, Copenhagen, Denmark). Briefly, a three-step peroxidase method was performed according to the manufacturer’s instruction. PC-10 monoclonal antibody (Santa Cruz Biotechnology) was used at a dilution of 1:50, for 15 minutes at room temperature. The sections were counterstained with hematoxylin. Evaluation of PC-10 immunostaining was performed based on the percentage of positive nuclei of 400–600 hepatocytes from 4–6 highest positive fields at high power (400X), and was expressed as PCNA labeling index.

### Statistical Analysis

All data are expressed as mean ± standard error of the mean (SEM). Data were analyzed with a one-way analysis of variance with subsequent Student-Newman-Keuls test. Differences were considered significant when *P* < 0.05.

## Results

### The level of hepatic expression of CXC chemokines inversely impacts hepatocyte proliferation

**Fig 1:**
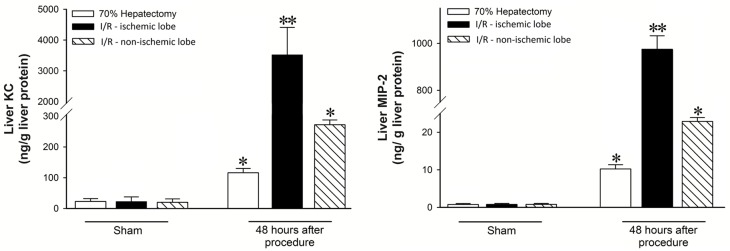
Differential hepatic expression of CXC chemokines in I/R injury and hepatectomy. Expression of MIP-2 and KC in the remnant liver after partial hepatectomy or ischemic and non-ischemic lobes after I/R injury. Data are mean ± SEM with n = 3–6 per group. *P<0.05 compared to sham group. **P<0.05 compared to ischemic liver.

Because it has been previously shown that the roles of CXC chemokines in the regenerative responses after hepatic I/R injury and partial hepatectomy are opposite in nature, we examined the tissue expression of CXC chemokines after hepatectomy and I/R to see if levels of these mediators were related to the observed biological effects. We examined chemokines 48 hours after the insult, as this is a time in both models when hepatocytes begin to actively proliferate. We found that levels of MIP-2 and KC in the livers of mice undergoing partial hepatectomy were significantly increased several fold, compared to sham mice (Fig. 1). However, after I/R injury, expression of MIP-2 and KC proteins in the ischemic lobes were increased hundreds- to thousands-fold over control (Fig. 1). These findings are consistent with the concept of relative “low” and “high” ligand concentrations, respectively.

To further examine this idea, we evaluated MIP-2 and KC levels in ischemic and non-ischemic lobes of the liver after I/R injury. We have previously shown that there is little injury or inflammation in non-ischemic liver lobes, but increased mRNA expression for inflammatory mediators [12]. MIP-2 and KC protein levels in ischemic liver lobes were increased thousands-fold, compared to sham (Fig. 1). Interestingly, expression of MIP-2 and KC in non-ischemic lobes was increased significantly compared to control (Fig. 1), but was significantly lower than ischemic lobes and much more similar to levels found in the liver after partial hepatectomy (Fig. 1).

**Fig 2:**
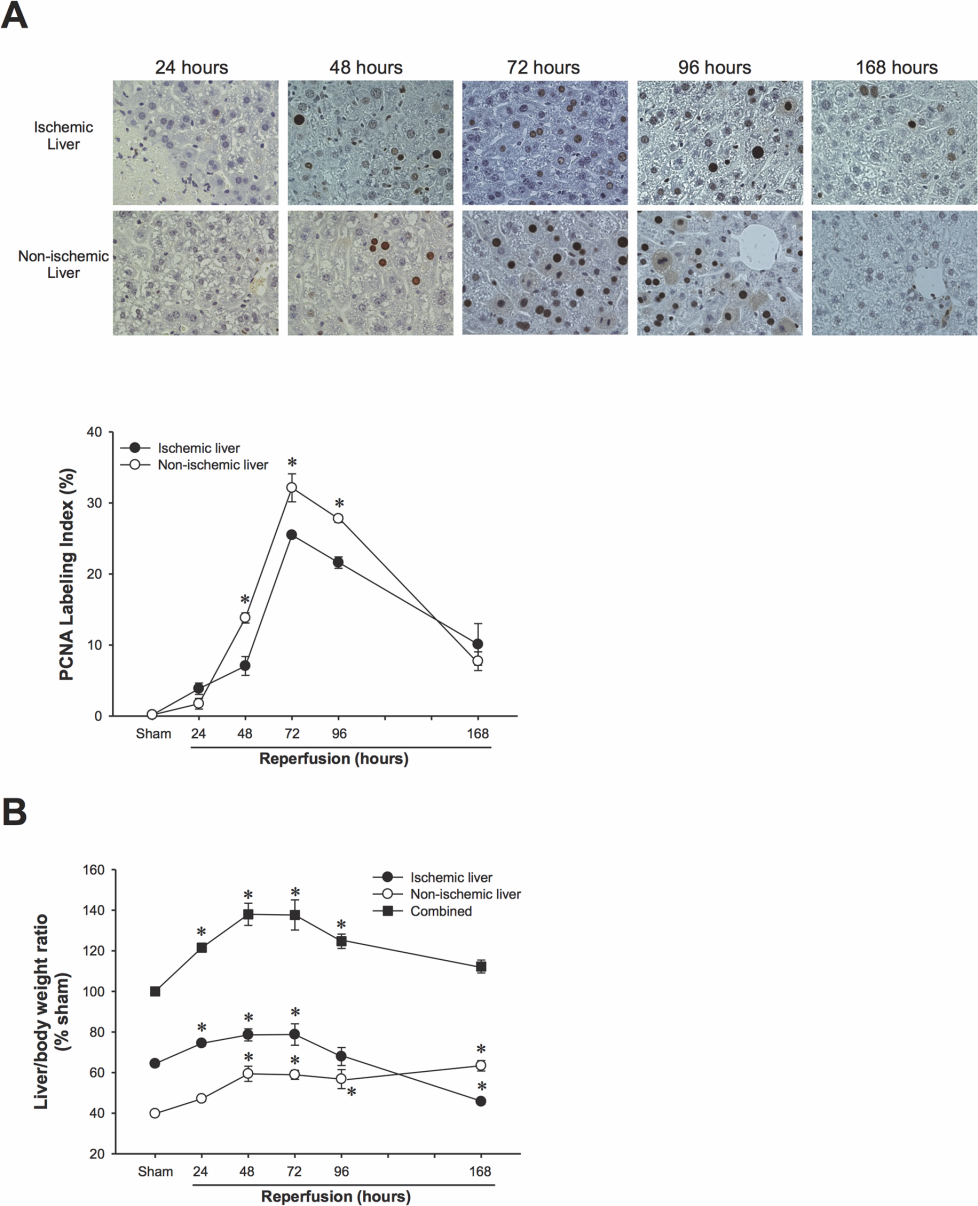
Hepatocyte proliferation and liver regeneration in ischemic and non-ischemic liver lobes after I/R injury. (A) Hepatocyte proliferation was determined by immunohistochemical staining for proliferating cell nuclear antigen (PCNA) and quantitative analysis of PCNA labeling. Data are mean ± SEM with n = 3–5 per group. *P<0.05 compared to ischemic liver. (B) Liver mass of ischemic and non-ischemic lobes after I/R injury. Data are mean ± SEM with n = 3–5 per group. *P<0.05 compared to sham.

Because we found a marked difference in CXC chemokine expression in the ischemic vs. non-ischemic lobes of the liver, we next evaluated hepatocyte proliferation in the different lobes. The time course of hepatocyte proliferation and liver regeneration was similar in both ischemic and non-ischemic lobes (Fig. 2). However, there was significantly more hepatocyte proliferation in non-ischemic lobes compared to ischemic lobes (Fig. 2A). Importantly, this translated into notable differences in the mass of the non-ischemic vs ischemic lobes. Non-ischemic lobes showed a significant increase in mass, while ischemic lobes showed a significant decrease in mass (Fig. 2B).

### CXC chemokines mediate hepatocyte cell death or proliferation in vivo via CXCR2

**Fig 3:**
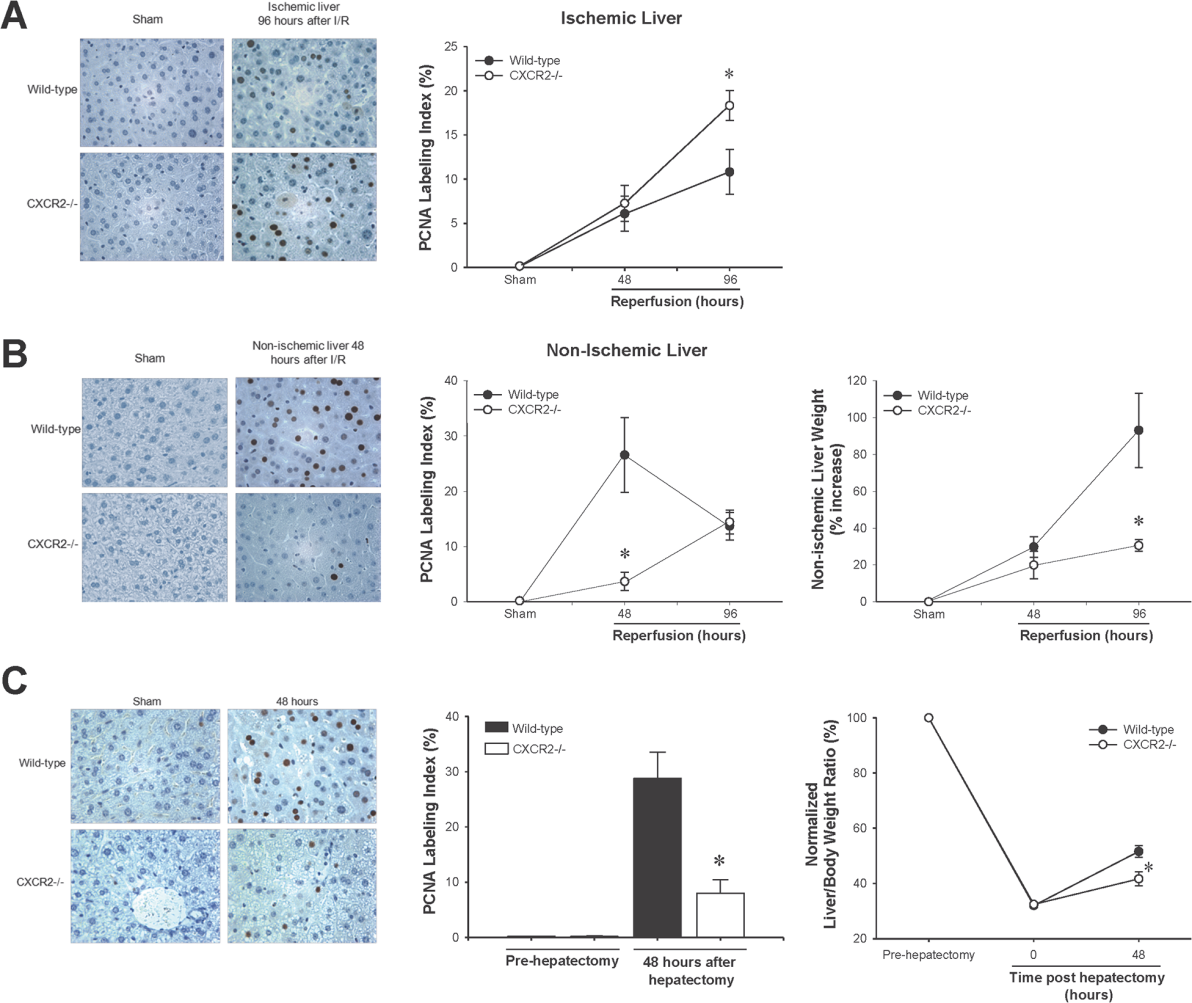
Effect of CXCR2 on hepatocyte proliferation and liver regeneration after I/R injury (A) and partial hepatectomy (B) was determined by immunohistochemical staining for proliferating cell nuclear antigen (PCNA) and quantitative analysis of PCNA labeling. (A) Hepatocyte proliferation in post-ischemic liver (upper panel) after I/R injury showed a significant increase in CXCR2−/− mice. Data are mean ± SEM with n = 3–14 per group. *P<0.05 compared to wild-type mice. Hepatocyte proliferation and liver regeneration in non-ischemic liver (lower panel) after I/R injury was significantly reduced in CXCR2−/− mice. Data are mean ± SEM with n = 3–6 per group. *P<0.05 compared to wild-type mice. (B) Hepatocyte proliferation and liver regeneration after partial hepatectomy showed a significant decrease in CXCR2−/− mice. Data are mean ± SEM with n = 3–4 per group. *P<0.05 compared to wild-type mice.

Because we have previously shown that CXCR2 is the primary receptor that mediates the effects of CXC chemokines on liver recovery and regeneration in ischemic liver lobes after I/R [5,8], we next examined the role of CXCR2 in hepatocyte proliferation in ischemic vs. non-ischemic lobes. Similar to our previous studies, we found that hepatocyte proliferation in the ischemic liver lobes from CXCR2-knockout mice was significantly increased above those from wild-type control mice (Fig. 3A). In stark contrast, hepatocyte proliferation was markedly decreased in non-ischemic liver lobes from CXCR2-knockout mice compared to those from wild-type controls (Fig. 3B). These effects were accompanied by a significant reduction in the growth of liver mass of the non-ischemic lobes in CXCR2-knockout mice compared to wild-type mice (Fig. 3B). Likewise, when we examined the role of CXCR2 in hepatocyte proliferation and growth of liver mass after partial hepatectomy, we found similar effects; knockout of CXCR2 significantly reduced hepatocyte proliferation and functional liver mass compared to wild-type controls (Fig. 3C).

### CXC chemokines function as a rheostat for hepatocyte proliferation and liver regeneration

**Fig 4:**
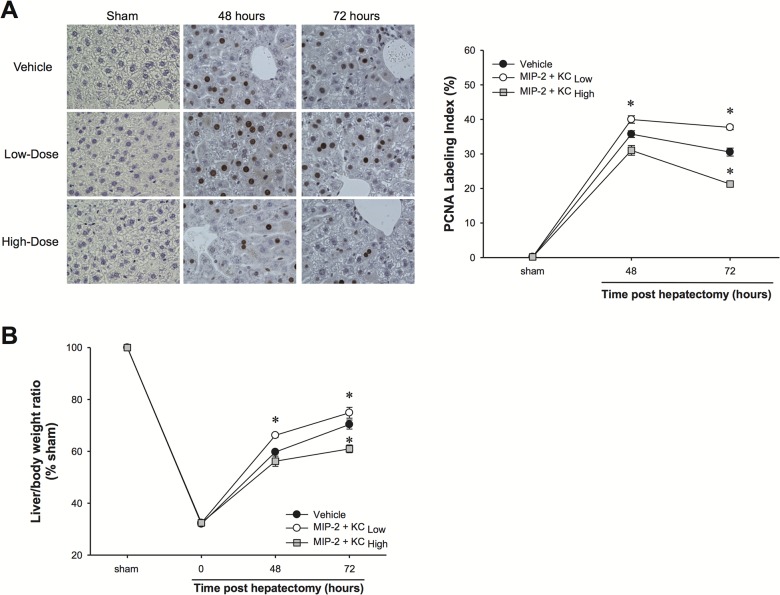
Effects of exogenous MIP-2 and KC treatment on hepatocyte proliferation and liver regeneration after partial hepatectomy. Wild-type mice were injected intravenously with high doses or low doses of MIP-2 and KC, starting 24 hours after hepatectomy and continued daily. An identical volume of sterile phosphate-buffered saline (PBS) was used as a vehicle control. (A) Hepatocyte proliferation was determined by immunohistochemical staining for proliferating cell nuclear antigen (PCNA) and quantitative analysis of PCNA labeling. Data are mean ± SEM with n = 4–8 per group. *P<0.05 compared to vehicle group. Original magnification was 400X. (B) Liver regeneration was determined by increases in liver mass. Data are mean ± SEM with n = 4–8 per group. *P<0.05 compared to vehicle group.

Thus far, our data suggest that locally expressed concentrations of CXC chemokines dictate the hepatocyte response (cell death vs. proliferation) in vivo. To directly test whether CXC chemokines function as a biological rheostat for hepatocyte proliferation and liver regeneration, we subjected mice to partial hepatectomy followed 24 hours later with exogenous administration of MIP-2 and KC. While previous studies have shown that exogenously administered MIP-2 promotes liver regeneration in this model [6,11], the doses used in those studies were low, relative to what we have observed after I/R injury. Therefore, we established doses of MIP-2 and KC that replicated the serum levels of these chemokines observed 48 hours after I/R injury (data not shown); this represented the “high” dose. The “low” dose was determined by multiplying the “high” dose by the ratio of observed tissue levels of MIP-2 and KC 48 hours after hepatectomy vs I/R (“low” dose = “high” dose x $\frac{\text{observed hepatectomy MIP-2 and KC}}{\text{observed I/R MIP-2 and KC}}$). Phosphate-buffered saline was used as a vehicle control. Vehicle-treated mice showed typical hepatocyte proliferation and liver regeneration after partial hepatectomy (Fig. 4A and 4B, respectively). Treatment with “low” doses of MIP-2 and KC significantly increased hepatocyte proliferation and regeneration. In contrast, treatment with “high” doses of MIP-2 and KC, resulted in a significant reduction in hepatocyte proliferation and liver regeneration (Fig. 4A and B, respectively).

### CXC chemokines do not promote hepatocyte proliferation in the normal liver

**Fig 5:**
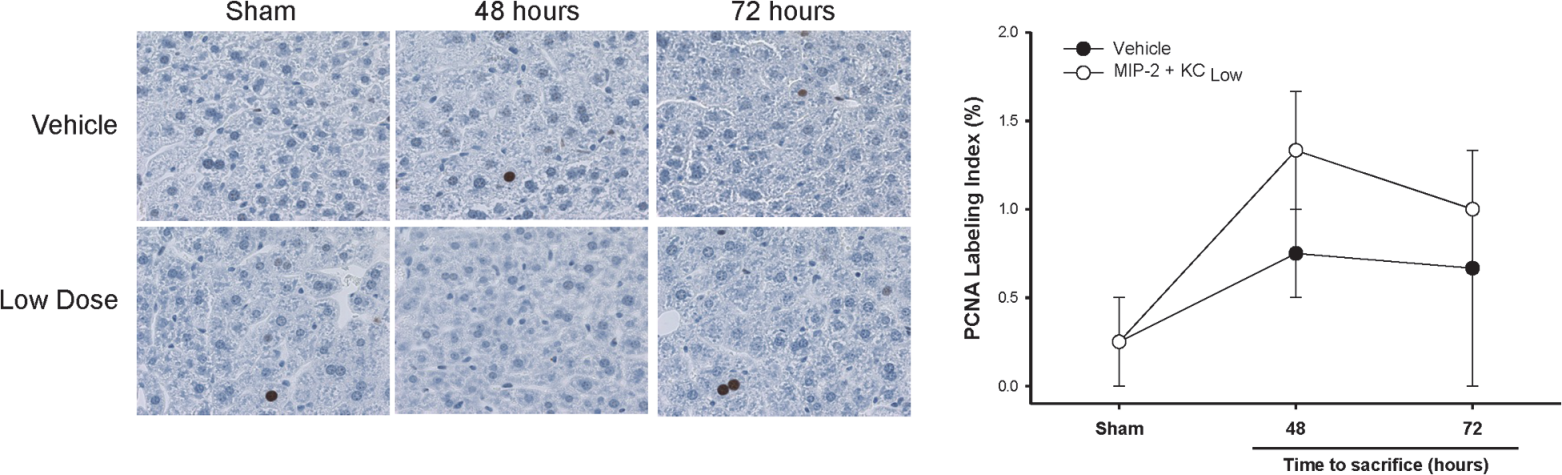
Effects of exogenous MIP-2 and KC treatment on hepatocyte proliferation in the normal liver. Wild-type mice were injected intravenously with low doses of MIP-2 and KC every 24 hours. An identical volume of sterile phosphate-buffered saline (PBS) was used as a vehicle control. Hepatocyte proliferation was determined by immunohistochemical staining for proliferating cell nuclear antigen (PCNA) and quantitative analysis of PCNA labeling. Data are mean ± SEM with n = 4–8 per group. Original magnification was 400X.

Because our data demonstrate that low doses of MIP-2 and KC caused hepatocyte proliferation after partial hepatectomy and are associated with increased hepatocyte proliferation in non-ischemic lobes after I/R injury (Figs. 1–4), we next assessed if low doses of MIP-2 and KC would stimulate hepatocyte proliferation in the normal, unstressed liver. Mice were injected intravenously with low dose MIP-2 (0.0002 ng/g) and KC (0.006 ng/g) at time 0 and then every 24 hours thereafter. As shown in Fig. 5, low dose chemokine treatment had no effect on hepatocyte proliferation in the normal liver.

## Discussion

The current study extends our previous work and provides several new insights. First, our data, combined with the observations of other laboratories, suggest that the dose-effects of CXC chemokines on liver regeneration are not related to hepatocyte stress. We have previously posited that the stress level of hepatocytes may alter their responsiveness towards CXC chemokines. For example, after partial hepatectomy, the remaining hepatocytes are relatively normal, whereas after I/R the hepatocytes are significantly stressed by the oxidative and inflammatory milieu. However, in the present studies we have demonstrated that exogenous administration of CXC chemokines after partial hepatectomy, at doses that replicate levels found after I/R injury, resulted in reduced hepatocyte proliferation and liver mass. Similar findings were observed by Stefanovic et al. who showed that adenoviral-mediated overexpression of KC in otherwise normal murine liver resulted in massive hepatocyte necrosis and liver dysfunction [13]. Conversely, in a model of acetaminophen toxicity, relatively low expression levels of CXC chemokines have been associated with improved liver regeneration [7]. In this latter model, hepatocytes are under significant oxidative/toxic stress, yet low expression of CXC chemokines (relative to I/R) promotes hepatocyte proliferation and liver regeneration. Collectively, these studies suggest that the dose-dependent effects of CXC chemokines on liver repair and regeneration are unrelated to hepatocyte stress.

Another important observation of the present studies relates to the important role for CXC chemokines and their receptor, CXCR2, in the homeostatic mechanisms regulating liver repair and recovery after I/R injury. While we have previously reported on this relationship in the post-ischemic liver [8], our current work demonstrates that CXC chemokines are critical for the compensatory increase in hepatocyte proliferation and increase in liver mass of non-ischemic lobes of the liver. Most interesting was our observation that this occurs in the presence of concentrations of CXC chemokines that are similar to those found in liver post-hepatectomy. The proliferative effects of CXC chemokines in non-ischemic liver lobes was highly dependent upon the expression of CXCR2, as gene deletion of this receptor abrogated the increases in both hepatocyte proliferation as well as liver mass. These findings provide more evidence of the global role of CXC chemokines in the processes of liver repair and regeneration regardless of the insult.

The manner in which CXC chemokines signal in hepatocytes, to modulate cell proliferation or cell death, is not yet known. To date, hepatocyte growth factor (HGF), epidermal growth factor (EGF), and transforming growth factor α (TGFα) have been the only identified primary mitogens for hepatocytes in culture—inducing clonal expansion of hepatocytes in the absence of serum factors [14]. Previous work from our laboratory has suggested that CXC chemokines do not induce proliferation in the absence of serum [6,8,15]. This suggests that CXC chemokines function by regulating the hepatocyte response to direct mitogens, and do so in a dose-dependent fashion. Thus, CXC chemokines appear to serve as critical regulatory factors that provide a secondary layer of control for liver repair and regeneration, by augmenting or limiting the effects of direct mitogens. Furthermore, our current data demonstrate that, in vivo, addition of low amounts of CXC chemokines shown to promote proliferation after partial hepatectomy, do not alter hepatocyte proliferation in the normal liver. This underscores the concept that CXC chemokines represent a secondary level of regulation of hepatocyte proliferation that is operant only under the appropriate conditions, such as after loss of functional mass due to injury or surgery.

In summary, the present study demonstrates that high concentrations of CXC chemokines within the liver are detrimental to liver repair and regeneration. In contrast, lower levels of these mediators promote hepatocyte proliferation and recovery of functional liver mass. Our results suggest that clinical management of hepatic levels of CXC chemokines may be an important consideration in patients recovering from acute liver injury, liver resection or transplantation.

## Supporting Information

S1 Dataset Datasets for Figs 1–5. Datasets for individual figures are in individual tabs of the spreadsheet.
(XLSX)
